# Genetic Risk Factors for Alzheimer's Disease in Racial/Ethnic Minority Populations in the U.S.: A Scoping Review

**DOI:** 10.3389/fpubh.2021.784958

**Published:** 2021-12-24

**Authors:** Lindsey Rubin, Lucy A. Ingram, Nicholas V. Resciniti, Brianna Ashford-Carroll, Katherine Henrietta Leith, Aubrey Rose, Stephanie Ureña, Quentin McCollum, Daniela B. Friedman

**Affiliations:** ^1^Department of Health Promotion, Education, and Behavior, University of South Carolina, Columbia, SC, United States; ^2^Department of Epidemiology and Biostatistics, University of South Carolina, Columbia, SC, United States; ^3^School of Medicine, University of South Carolina, Columbia, SC, United States; ^4^College of Social Work, University of South Carolina, Columbia, SC, United States

**Keywords:** genetic risk factors, Alzheimer's disease, race, ethnicity, minority, review

## Abstract

**Objectives:** As the United States (U.S.) population rapidly ages, the incidence of Alzheimer's Disease and Related Dementias (ADRDs) is rising, with racial/ethnic minorities affected at disproportionate rates. Much research has been undertaken to test, sequence, and analyze genetic risk factors for ADRDs in Caucasian populations, but comparatively little has been done with racial/ethnic minority populations. We conducted a scoping review to examine the nature and extent of the research that has been published about the genetic factors of ADRDs among racial/ethnic minorities in the U.S.

**Design:** Using an established scoping review methodological framework, we searched electronic databases for articles describing peer-reviewed empirical studies or Genome-Wide Association Studies that had been published 2005–2018 and focused on ADRD-related genes or genetic factors among underrepresented racial/ethnic minority population in the U.S.

**Results:** Sixty-six articles met the inclusion criteria for full text review. Well-established ADRD genetic risk factors for Caucasian populations including *APOE, APP, PSEN1*, and *PSEN2* have not been studied to the same degree in minority U.S. populations. Compared to the amount of research that has been conducted with Caucasian populations in the U.S., racial/ethnic minority communities are underrepresented.

**Conclusion:** Given the projected growth of the aging population and incidence of ADRDs, particularly among racial/ethnic minorities, increased focus on this important segment of the population is warranted. Our review can aid researchers in developing fundamental research questions to determine the role that ADRD risk genes play in the heavier burden of ADRDs in racial/ethnic minority populations.

## Introduction

As the United States (U.S.) population rapidly ages, the incidence of Alzheimer's Disease and related dementias (ADRD) is on the rise (1, 2). Alzheimer's Disease (AD) is the sixth leading cause of death in the U.S. and the fifth leading cause of death for those age 65 years and older (1, 2). In the U.S., 5.7 million people are living with AD, which is projected to grow to 13.9 million adults (3.3% of the population) by 2060 (2). Although the primary risk factor for ADRD is age, race, and ethnicity are also associated with ADRD (2–4).

The U.S. population is becoming more racially and ethnically diverse, with Census projections showing that the country will be a “majority–minority” nation by 2050. That is, racial/ethnic minorities will comprise more than 50% of the population by this date (5). African Americans are twice as likely as Non-Hispanic Whites to have AD, while Hispanics are 1.5 times as likely to have AD compared to their Non-Hispanic White counterparts (1). Also by 2050 in the U.S., it is estimated that the proportion of racial/ethnic minorities who suffer from AD will double in size compared to current figures (6). Regarding rates of diagnoses, in particular, African Americans are diagnosed later in the course of ADRD than White patients. Quinones et al. (7) suggest that this is likely due to cultural factors and normalization of ADRD symptoms as part of the usual aging process. There are also noted disparities in cognitive decline and impairment with racial/ethnic minorities suffering greater cognitive decline after ADRD diagnosis compared to other groups (8–10), potentially related to socio-economic resources, such as education quality, development of cognitive reserve, financial means, and early midlife stressors (7). Racial/ethnic health disparities in the U.S. proliferate, are multilayered, and are rooted in a variety of structural and historical inequalities that continue to disproportionately burden racial/ethnic minorities. These disparities underscore the need to examine the factors underlying ADRD in racial/ethnic minority U.S. populations.

As our population ages and the size of our minority populations increase in the U.S., understanding the burden of ADRD on our aging populations can aid in providing insight into the most appropriate and effective public health actions. For example, to provide the best care and community support for aging minority populations, it is valuable to understand any patterns of genetic risk factors to address comorbid disease management, environmental, and socio-economic factors that may affect ADRD prevention, diagnosis, and progression. Similarly, more precise knowledge of differences in prevalence of ADRD in minority populations is useful for policy planning when allocating resources, ensuring access to care, and improving quality of care (11).

Much research has been undertaken to test, sequence, and analyze genetic risk factors for ADRD in White populations, but comparatively little has been done with racial/ethnic minority populations (12). In fact, examining genetic factors in heath disparities research has sometimes led to intense controversy (13, 14), oftentimes for concern of the racialization of medicine, misuse of pharmacogenomics, and racial biology (15–17). In studies that have explored ADRD genetic risk factors in minorities, the study sizes have been relatively small, making the conclusions about genetic associations less powerful. Some data appears to show differences in the genetic etiologies between Caucasians and African Americans, especially relating to the *APOE* gene, which needs to be explored further (11, 18, 19).

There are multiple types of AD classified by age at onset and method of inheritance. The two main categories from a genetic perspective are Early Onset Alzheimer's Disease (EOAD) and Late Onset Alzheimer's Disease (LOAD). According to the National Institute on Aging website, EOAD is also referred to as Familial Alzheimer's Disease and follows an autosomal dominant inheritance pattern, meaning that only one allele from either parent is required to cause disease. EOAD is caused by mutations in three genetic loci, *APP, PSEN1*, and *PSEN2* (20–22). Late Onset Alzheimer's Disease, which is also referred to as Sporadic Alzheimer's Disease, is polygenic, meaning that multiple genes along with environmental factors contribute to the risk of AD, age of onset, and severity of disease (20, 21). *APOE* is one of the most well-established genetic risk factors for LOAD and has implications for risk of other types of AD (20, 21).

The *APOE* e4 allele is a strong risk factor for Sporadic or Late-Onset Familial AD, with the degree of risk increased with two copies of the allele (homozygous e4/e4), but possession of an e4 allele is not in itself necessary to produce AD or sufficient alone to cause the disease (23). Homozygosity in genetics refers to an individual with two copies of the same allele at a particular genetic loci or gene, while heterozygosity refers to the presence of two different alleles at a loci or gene (24). The effects of homozygosity and heterozygosity for the e4 allele has been studied extensively in European American populations, with homozygotes having a 12 times increased risk of LOAD, and heterozygotes having a 2–3 times increased risk of LOAD (18, 19). In African American populations and Hispanic populations, e4 heterozygosity or homozygosity does not correlate with increased risk of AD, indicating that other genetic and environmental factors are responsible for the increased incidence and prevalence of AD in these populations (25–27).

Examining genetic risk factors for ADRDs in minority populations can deepen our understanding of the interaction between biological or genetic factors and socio-ecological determinants of health. It also has the potential to aid in preventive care and early diagnosis for these populations with greater incidence of ADRDs (28). To better understand the risk profile of racial/ethnic minorities who are impacted by ADRD, research should be conducted to comprehend the disease mechanism in these populations, including influential genetic risk factors. If advances in genomic medicine continue to be valid, reliable, and promising, racial/ethnic minorities should be afforded the opportunities to participate in research at similar rates as their White counterparts (13). Other systematic reviews have been conducted in this general subject area. These reviews have had a more segmented focus, with some examining one specific gene and others focusing on a specific population (29, 30). Additional scoping or systematic reviews were focused on a single type of ADRD, such as Lewy Body Dementia or LOAD (31, 32). To explore this gap in the literature, we conducted a scoping review to examine the nature and extent of research that has been published about the genetic factors of ADRDs among racial/ethnic minorities in the U.S.

## Methods

### Search Strategy and Selection Criteria

Our study protocol was developed using the established and peer-reviewed scoping review methodological framework and updated based on prior ADRD-focused scoping reviews (33–38). Scoping reviews are a useful format used to explore fields of study not already well-explored or defined. A scoping review is a “preliminary assessment of the potential size and scope of available research literature. It aims to identify the nature and extent of research evidence” [(39), p. 31]. Scoping reviews can be utilized for a variety of research purposes including discovering the scope of existing research in a field of study, in order to identify gaps in the literature for future study. Scoping reviews can also be used to explore the need for a systematic review and the potential value of a systematic review (34, 38).

The databases used to conduct the search were PubMed, CINAHL, and Science Direct. We chose to limit the search to those articles published from 2005 to 2019, as 2005 is when next generation DNA sequencing was available, allowing for more extensive genetic studies with larger sample sizes (40). We conducted a search within the databases using a combination of three concepts: (1) ADRD Genes, (2) Populations and Minority Groups, and (3) ADRDs. The search used a combination of terms from the three concepts to find articles relevant to our research questions. Specific ADRD candidate gene terms were chosen by recent data from Genome Wide Association Studies (GWAS) (41, 42). Some included terms were: *APOE*, beta Amyloid Protein Precursor, *CD2AP*, Genetic Predisposition to Disease, *PSEN1, PSEN2, STM2, APP, TREM2*, African American, Alaska Native, Arabs, Asian American, Ethnic Groups, Hispanic American, Native American, Jews, Minority Groups, Alzheimer's Disease, Dementia, Lewy Bodies, Lewy Body Disease. Inclusion criteria for the review were (1) articles published after January 1, 2005, (3) available in English, (3) peer reviewed empirical studies or Genome-Wide Association Studies (GWAS) (4) that focus on or include an underrepresented minority population in the U.S., (5) that focus on ADRDs, and (6) that focus on ADRD-related genes or genetic factors.

### Data Extraction and Synthesis

The study selection process included three interrelated steps: Title/abstract reviews, full-article reviews, and reviewers' examination of reference lists from full articles to identify articles for possible inclusion (43). First, five out of nine of our team members were randomly assigned to review the 1,134 article titles and abstracts in Covidence systematic review online software, with each abstract randomly assigned to two reviewers. Two team members were designated as arbitrators for review discrepancies. When a discrepancy occurred between reviewers (e.g., one “Yes, include in the review” and one “No, do not include in the review”), the designated team members arbitrated the discrepancy. When both randomly assigned reviewers marked an abstract as “Yes” for inclusion, Covidence automatically moved it into the full article review list. Once all titles and abstracts were reviewed twice and all discrepancies arbitrated, the research team then performed a complete review of the resulting 115 articles. Seven team members were randomly assigned a set of articles for full review and the same inclusion and exclusion criteria were used. A data abstraction tool was developed to facilitate review of the full articles and to abstract relevant data. The tool included 21 questions to aid in summarizing the key characteristics of each article. Discrepancies on final article selection and data extraction were then arbitrated by two team members with consultation with the rest of the research team. Once all full articles had been determined, the abstracted data were converted to a Microsoft Excel file for management.

## Results

### Studies Identified

**Figure 1:**
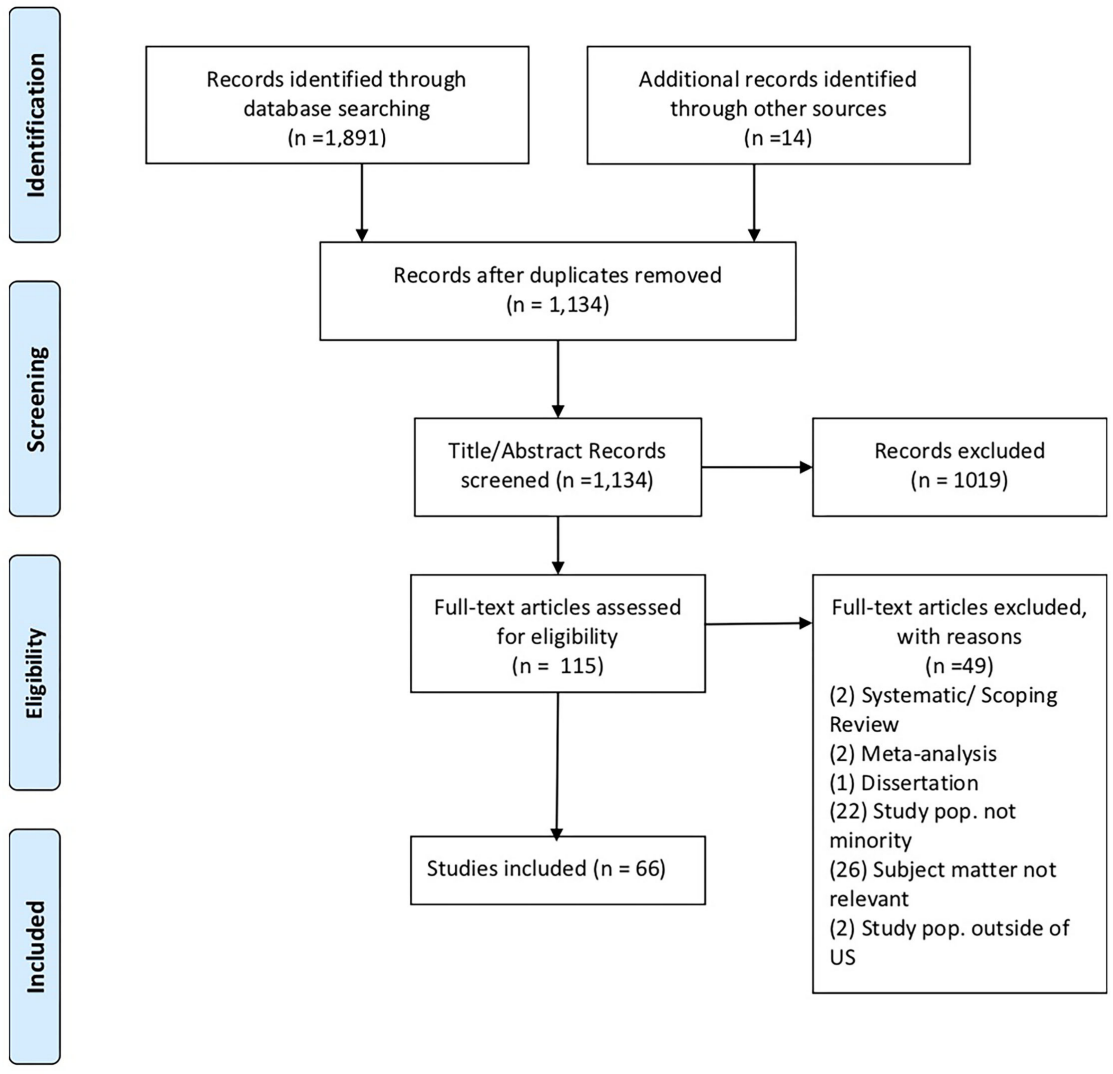
PRISMA chart. Source: Moher et al. (43).

From the searches in all three databases there were a total of 1,891 articles and 14 additional articles identified from reference lists, for a total of 1,905. We removed 771 duplicates, for a total 1,134 articles for the abstract review stage. During the title abstract review we excluded 1,019 articles due to the following reasons: published outside of the date range, article not available in English, dissertation, metanalysis, systematic review, scoping review, not focused on ADRD, not focused on minority U.S. population, not focused on ADRD genetic factors. After title abstract review, 115 articles remained for full text review. An additional 49 articles were excluded during the full-text review stage if the criteria were not met through examination of the full article. The full text review resulted in 66 included articles (see Figure 1).

### Populations and Genes Examined

Tables 1, 2 present the general characteristics of the studies included in the full-text review. Table 3 presents a detailed listing of the characteristics of the articles that were included in the full-text review. Among the resulting 66 studies, most of the studies (*n* = 41, 62%) were focused on African Americans as the population of interest followed by those focusing on the Hispanic population (*n* = 28, 42%). Asian American populations were examined in seven out of the 66 studies (11%), and Native American/Alaska Natives populations were included in only one study (1.5%) (Table 1).

**Table 1:** Characteristics of studies included in the full-text review (*N* = 66).

**Characteristic**	**Number**	**Percentage (%)**
**Publication year**		
2005–2006	7	10.6
2007–2008	6	9.1
2009–2010	5	7.6
2011–2012	9	13.6
2013–2014	15	22.7
2015–2016	9	13.6
2017–2018	15	22.7
**Race/Ethnicity** *^a^*		
African American	41	62.1
Hispanic American	28	42.4
Asian American	7	10.6
Native American/Alaska Native	1	1.5
**Sample size**		
0–100	3	4.5
101–500	11	16.7
501–1,000	10	15.2
1,001–1,500	11	16.7
1,501–2,000	7	10.6
2,001–2,500	4	6.1
2,501–3,000	2	3.0
3,001–3,500	2	3.0
3,501–4,000	0	0.0
4,001–4,500	1	1.5
4,501 or more	15	22.7
**Type of study**		
Case-control	18	27.3
Cross-sectional	15	22.7
Cohort	12	18.2
Genome Wide Association Study (GWAS)	9	13.6
Longitudinal	5	7.6
Other	5	7.6
Case report/case study	2	3.0

a *Some articles included multiple races/ethnicities in the study sample*.

**Table 2:** Type of ADRD and risk genes identified in full-text review articles (*N* = 66).

**Characteristic**	**Number**	**Percentage (%)**
**Type of ADRD** *^a^*		
Lewy Body Dementia	1	1.5
Mild Cognitive Impairment	2	3.0
Cognitive Decline	2	3.0
Vascular Dementia	4	6.1
Early onset AD (EOAD)	7	10.6
Alzheimer's Disease	13	19.7
Type of ADRD not specified	20	30.3
Late Onset AD (LOAD)	26	39.4
**ADRD risk genes identified** *^b^*		
PSEN2	1	1.5
AKAP9	2	3.0
GRIN3B	2	3.0
SORL1	2	3.0
CR1	3	4.5
APP	4	6.1
PSEN1	4	6.1
ABCA7	6	9.1
CLU	6	9.1
PICALM	7	10.6
APOE	43	65.2
Other	42	63.6

a *Some articles examined more than one type of ADRD*.

b *Some articles included multiple risk genes*.

**Table 3:** Detailed listing of studies included in the full-text review.

**Author and year**	**Study design**	**URM group**	**Data source**	Sample size^*^	**Type of ADRD**	**Gene(s) included**
Akomolafe et al. (44)	Case-control	African American	MIRAGE Study	511 cases, 679 controls^*^	EOAD, LOAD	*NOS3, APOE*
Arnold et al. (45)	Cohort	Puerto Rican	Original data	283	EOAD, LOAD	*PSEN1*
Beeri et al. (46)	Longitudinal, cohort	African American	ACCORD-MIND Study	466	Cognitive Decline	*HP*
Borenstein et al. (47)	Prospective, cohort	Japanese American	The Kame Project	1,859	Alzheimer's disease	*APOE*
Borenstein et al. (48)	Prospective, cohort	Japanese American	The Kame Project	1,859	Vascular Dementia and Alzheimer's Disease	*APOE*
Bressler et al. (49)	GWAS	African Americans	The ARIC Study	10,359^*^	LOAD	*APOE, ABCA7, BIN1, CD2AP, CDS33, CELF1, EPHA1, MS4A4E, NME8, PICALM, PKT2B, ZCWPW1*,
Campos et al. (50)	Case-control	Hispanic Americans, Amerindians	Original data	56 cases, 56 controls^*^	Alzheimer's Disease	*APOE*
Carrion-Baralt et al. (51)	Cohort	Puerto Ricans	Original data	87	Alzheimer's Disease	*APOE*
Conway et al. (52)	Case-control, targeted sequencing	African Americans	Mayo Clinic	5,924 cases, 5,173 controls^*^	EOAD, LOAD, Lewy Body Dementia	*ABI3, APOE, PLCG2*
Cukier et al. (53)	Case-control	African Americans, Caribbean Hispanics	HIHG and ADGC data sets	149 cases, 137 controls^*^	LOAD	*ABC1, ABCA7*
Desai et al. (54)	Case-control	African Americans	ADRC data set	1,059 cases, 716 controls^*^	LOAD	*BDNF*
Edwards-Lee et al. (55)	Family study	African Americans	Original data	7	EOAD (autosomal dominant)	*APP, PS1, MAPT*
Erlich et al. (56)	Case-control study	African Americans	MIRAGE Study	520 cases, 677 controls^*^	Alzheimer's Disease	*PON1, PON2, PON3*
Fitten et al. (57)	Cross-sectional study	Hispanic Americans	ADRC data set, OVMC data set	290^*^	Alzheimer's Disease, Vascular Dementia	*APOE*
Ghani et al. (58)	Case-control, GWAS	Hispanic Americans	Washington Heights-Inwood Columbia Aging Project, Estudio Familiar de Influencia Genetica de Alzheimer Study	547 cases, 542 controls^*^	LOAD	*APOE, CLU, PICALM, BIN1*
Gonzalez et al. (59)	Cohort study	Hispanic Americans	The Hispanic Community Health Study/Study of Latinos (HCHS/SOL)	10,887^*^	Alzheimer's Disease	*APOE*
Harwood et al. (60)	Cross-sectional study	African Americans, Hispanic Americans	Original data	685	Alzheimer's Disease	*APOE*
He et al. (61)	Cross-sectional study	African Americans, Hispanic Americans	Original data	439	Mild Cognitive Impairment (MCI)	*APOE*
Hendrie et al. (62)	Case-control study	African Americans	Original data	221 cases, 218 controls	MCI, Dementia, Alzheimer's Disease	*APOE*
Hohman et al. (63)	Case-control, GWAS	African Americans	ADGC	1,840 cases, 3,804 controls	LOAD	*APOE, STM2, ABCA7, CR1, PICALM, BIN1, EPHA1, CD33, SLC24A4, GRIN3B, FERMT2, MS4A6A*
Janicki et al. (64)	Cohort study	African Americans, Hispanic Americans	Washington Heights Inwood Columbia Aging Project (WHICAP)	1,686^*^	Alzheimer's Disease	*APOE, CYP19*
Jin et al. (65)	Case-control	African Americans	Knight-ADRC + NIA-LOAD, Mayo Clinic, Indiana University, WHICAP, Emory University	906 cases, 2,487 controls^*^	LOAD	*TREM2*
Janicki et al. (66)	Prospective Cohort study	African Americans, Hispanic Americans	WHICAP	1,686^*^	Alzheimer's Disease	*ESR1*
Kim et al. (67)	Longitudinal prospective community-based study	African Americans	IIDP	1,858^*^	AD, Dementia	*CD2AP, CBS, DTWD2, DYNC111, JRKL-AS1, BIRC8, HCY*
Kuller et al. (68)	Longitudinal cohort study	African Americans	Pittsburgh Cardiovascular Health Study	532	LOAD	*APOE*
Kunkle et al. (69)	Case-control study, GWAS	African Americans	HIHG/CWRU, NIMH Genetic Studies of Alzheimer's Disease Cohort, NCRAD/NIA-LOAD, African American Alzheimer's Disease Genomics Coalition (AAADGC)	2,762 cases, 2,812 controls^*^	LOAD	*ABCA7*
Kwon et al. (70)	Cohort study	African Americans, Hispanic Americans	Original data	1,309^*^	LOAD	*APOE*
Lee et al. (71)	Nested Case-control study, prospective	African Americans, Hispanic Americans	Original data	296 cases, 428 controls^*^	AD	*SORL1*
Lee et al. (72)	Family-based cohort study, GWAS	Caribbean Hispanic	Original data	1,161 individuals from 209 families	Familial LOAD	*APOE, PSEN1*, 5q15, 7q36.3, 14q32.12, 17q25.1, 17p13
Lee (73)	Family-based case-control and unrelated case-control study, GWAS	Caribbean Hispanics	ADRC	693 cases, 442 controls^*^	LOAD	*APOE*, 12p13
Lee (74)	Nested case-control GWAS	Caribbean Hispanics	WHICAP and EFIGA datasets	549 cases, 544 controls	LOAD	*CLU, PICALM, BIN1, PSEN1, GHITM, C10orf99, PCDH21, LRT2, LRT1, RGR, DGKB, HPCAL1, ODC1*
Lee (75)	Family-based cohort study	Caribbean Hispanics	WHICAP and EFIGA datasets	2,888	EOAD, LOAD	*PSEN1, SNX25, PDLIM3, SORBS2, SH3RF3, NPHP1*
Livney (76)	Cross-sectional study	African American, Hispanic Americans	Original data	1,341	AD	*APOE*
Logue (77)	Case-control study	African Americans	MIRAGE, GenerAAtions, ADNI, GenADA, NIA-LOAD, FHS	3,568 cases, 6,205 controls^*^		*APOE, PVRL2, CLU, PICALM, BIN1, EPHA1, MS4A, ABCA7, and CD33, TOMM40*
Logue et al. (78)	Case-control	African Americans	MIRAGE Study, GenerAAtions Study	422 cases, 394 controls	EOAD, LOAD	*AKAP9, APOE, BIN1, CLU, CR1, PICALM, MS4A6E, CD2AP, CD33, ABCA7, EPHA1, SORL1, ACE, PSEN1, PSEN2, APP*
Logue et al. (79)	Case-control	African Americans	MIRAGE Study, GenerAAtions Study, National Cell Repository for Alzheimer's Disease (NCRAD), Ibadan/Indianapolis (INDY) Study	489 cases, 472 controls	LOAD	*ABCA7, AKAP9, KIAA0196, KANSL1, CNN2, TRIM2*
Marden et al. (80)	Cohort	African Americans	Health and Retirement Study (HRS)	7,690^*^	AD and Dementia	*APOE, BIN1, CLU, ABCA7, CR1, PICALM, MS4A6A, CD33, MS4A4E, CD2AP*
Marden et al. (81)	Cohort	African Americans	HRS	8,253^*^	AD	*APOE, CLU, CR1, PICALM*
McAninch et al. (82)	Cohort	African Americans	Original data	12,348^*^	AD	*DIO2*
Melville et al. (83)	Case-control	African Americans	MIRAGE Study, ADNI Study	1,146 cases, 956 controls^*^	AD, MCI	*APOE, PICALM, F5/SELP, LHFP, GCFC2, SYNPR*, TTC27
Mez et al. (84)	Case-control	African Americans	ADGC, GenerAAtions, MIRAGE, CHAP	1,825 cases, 3,784 controls	LOAD	*APOE, ABCA7, COBL, SLC10A2*
Mount et al. (85)	Cross-sectional, retrospective	African Americans	ADCR	65	LOAD	*APOE*
Murrell et al. (86)	Cohort	African Americans	Original data	480	LOAD	*APOE*
N'Songo et al. (87)	Cohort	African Americans	Original data	198 cases, 350 controls	EOAD	*APP, PSEN1, PSEN2*
O'Bryant et al. (88)	Cohort	Mexican Americans	Project FRONTIER, TARCC	1,628	MCI	*APOE*
O'Bryant et al. (89)	Cohort, CBPR	Mexican Americans	Project FRONTIER, TARCC	1,069^*^	MCI, AD	*APOE*
Olarte et al. (90)	Population-based, case series	Hispanics	HCFA	680	Sporadic and familial AD	*APOE*
Pedraza et al. (91)	Case-control	African Americans	Mayo Clinic Alzheimer's Disease Research Center Data, Mayo Clinic Study of Aging, Mayo Clinic LOAD-GWAS	476 cases, 2,443 controls^*^	LOAD	*CLU, CR1, PICALM*
Peila et al. (92)	Nested case-control	Japanese-Americans	Honolulu-Asia Aging Study (HAAS), Honolulu Heart Program (HHP)	283 cases, 573 controls	AD, Vascular Dementia	*APOE, TGF-β1*
Petrovich et al. (93)	Longitudinal, cohort	Japanese-Americans	The Honolulu-Asia Aging Study	375	AD	*APOE*
Qian et al. (94)	Prospective, cohort	Latinos	NACC, Rotterdam Study, Framingham Heart Study, and Sacramento Area Latino Study (SALSA)	16,844^*^	AD	*APOE*
Rajabli et al. (95)	Case-control	African Americans, Hispanic Americans	HGDP (Human Genome Diversity Project)	1,986 cases, 3,899 controls^*^	LOAD	*APOE*
Reitz et al. (96)	Case-control	African Americans and Caribbean Hispanics	Toronto dataset, NIA-LOAD, MIRAGE Caucasian dataset, MIRAGE African American dataset, Miami Caucasian, Caribbean Hispanic dataset	2,809 cases, 3,482 controls	AD	*SORCS1, APP, A*β*, SORL1*
Reitz et al. (97)	Case-control	Caribbean Hispanics	DMS-IV, NINCDS-ADRDA	160 cases, 294 controls	LOAD	*IDE, KIF1, HHEX*
Reitz et al. (98)	Case-control	African Americans	CHAP, MARS/CORE, UM/VU	1,968 cases, 3,928 controls	LOAD	*ABCA7, APOE*
Rippon et al. (99)	Family-based cohort study	Latinos	NINDCS-ADRDA	1,498	Familial AD	*APOE*
Roses et al. (100)	Cohort	African Americans, Japanese Americans	Bryan ADRC Database/Repository, Coriell Cell Repositories	447^*^	LOAD	*TOMM40, APOE*
Sacyzynsky et al. (101)	Cohort	Japanese-Americans	The Honolulu Heart Program, Cooperative Lipoprotein Study	929	Dementia	*APOE*
Sawyer et al. (102)	Prospective cohort	African Americans	Duke EPESE Study	2,076^*^	Cognitive decline (CD)	*APOE*
Simino et al. (103)	Cohort	African Americans	CHARGE, the NHLBI Exome Sequencing Project	1,414^*^	AD	*Amyloid-*β, KLKB1, F12, PLIN2, ITPRIP
Tosto et al. (104)	Cohort	Caribbean Hispanics	NIA-LOAD, EFIGA	8,116^*^	LOAD	*APOE* ε4
Vardarajan et al. (105)	Case-control	African Americans	ADGC	8,309 cases, 7,366 controls^*^	AD	*APP, KIAA1033, SNX1, SORL1, SNX3, RAB7A*
Vardarajan et al. (106)	Family and cohort-based genetic association study	Caribbean Hispanics	Original data	464 familial subjects—(350 affected, 114 unaffected), 498 unrelated controls	LOAD	*SORL1*
Weiner et al. (107)	Case-control	Choctaw Indians	Original data (Choctaw Indians) and UT Southwestern Alzheimer's Disease Center (ADC)	78 cases, 39 controls^*^	AD	*APOE*
Yu et al. (108)	Longitudinal, cohort	African Americans	Religious Orders Study (ROS), Rush Memory and Aging Project (MAP), Minority Aging Research Study (MARS)	2,388^*^	AD	*APOE, TOMM40*

* *Article included multiple races/ethnicities in the study sample*.

There were many different study designs represented in our results. The most common study design was a case control study design, with 18 included articles using this design. The next most frequently found study design was cross-sectional with 15 included studies in this category. There were nine GWAS which is expected because candidate risk genes for ADRD in minority populations have not been fully established. There were five longitudinal studies in the results and two case studies. Lastly, there were five studies that could not be classified into one of these categories (Table 1).

Many different types of ADRDs were represented in our search results. The most frequently examined type of AD in our results was LOAD (*n* = 26, 40%), followed by AD (*n* = 12, 18%) and EOAD (*n* = 7, 11%). Vascular Dementia was the focus of four articles out of the total 66 results (*n* = 4, 6%). Both Mild Cognitive Impairment (MCI) and Cognitive Decline were examined in two articles each (*n* = 2, 3%). Lewy Body Dementia was the subject of one article (*n* = 1, 1.5%). Lastly, there were 20 articles that did not specify a particular ADRD designation (*n* = 20, 30%) (Table 2).

In terms of specific ADRD risk genes, *APOE* was examined in most studies, with 44 out of 66 included studies examining this genetic risk factor. Other potential ADRD risk genes that were examined by multiple studies included *ABCA7, CLU, CR1, PICALM, APP, PSEN1, SORL1* and *AKAP9, APP*, and *PSEN1* are well-established genetic risk factors for EOAD, but in total, they were examined in only eight out of 66 included studies (Table 2).

## Discussion

Our findings provide an overview of the published literature examining the association between genetic factors and ADRD risk among racial/ethnic minorities in the U.S. These findings help to illuminate knowledge gaps and suggest whether further study should be undertaken to assess more comprehensively the role that ADRD genes play in AD rates and disease outcomes for minority populations.

Regarding the extent of the genes examined in the studies that we found, *APOE* was examined in most studies, with 44 out of 66 included studies examining this genetic risk factor. This corresponds with extant ADRD genetic risk factor research findings in general, as *APOE* is the most well-established genetic risk factor for Sporadic or LOAD (23). We found that well-established ADRD genetic risk factors for Caucasian populations including *APOE, APP, PSEN1*, and *PSEN2* have not been studied to the same degree in minority U.S. populations. The *APOE* genotype has been shown to be less predictive of ADRD risk in African American, Asian American, Hispanic American, and Native American populations (26, 27, 29, 98). Other genetic risk factors may play a larger role in ADRD genetic risk in these populations, with potential candidates including genes with various functions such as *ABCA7, CLU, CR1, PICALM, SORL1, AKAP9*, and *TREM2* (26, 27, 29, 98, 109). These genes were noted in our review, however with far less frequency than *APOE*. Preliminary findings indicate that there may be a more complex polygenic profile of ADRD genetic risk in these populations, and this has potential implications for the possible polygenic nature of ADRD risk in all populations (27, 59, 87).

In comparison to the amount of research that has been conducted on Caucasians in the U.S., we found that some minority communities were vastly underrepresented in the research, namely Hispanics, Native Americans, and Asian Americans. Though the number of studies on ADRD genetic risk factors in minority populations has increased over time, especially for certain populations such as African Americans, more comprehensive studies with large sample sizes should be performed to establish key genetic risk factors for these populations as well (27, 109–112). Among the studies in our review, sample size for non-GWAS studies started as low as *N* = 19 for a case report design. As the sample size increases and more diverse persons are included, additional, more statistically sound conclusions can be made about the associations between genetic expression and disease outcome.

Additionally, comparative studies with both minority and majority population group samples would be useful in examining genetic risk factors, as well as the effects of environment and other factors. Studies exploring genetic risk factors in these populations is warranted to determine the role that both genes and environmental factors play in increased ADRD risks in these populations. A larger, systematic review of existing literature on genetic risk factors for minority U.S. populations would be an appropriate next step in better understanding the existing study landscape with intentions toward implementing GWAS and meta-analyses for diverse U.S. populations.

Knowledge gaps in the disease mechanism among racial/ethnic minority populations is a critical indicator of inequities in genetics and genomics research in these communities, as well as a lack of equity in the health care system for these groups (112). Advancements in genetic medicine and genomic research proliferate, unfortunately not at the same rate for all persons. The impact that disproportionate expansion, innovation, and progress in the field can have on health disparities is significant (12, 112). With that in mind, it is also important to acknowledge that while genetic inquiry is crucial to understanding the disparities present in ADRD, it is not the sole risk factor. Other factors such as environment and socio-environmental context, are implicated in the distribution of racial health disparities, and in fact, the complex interplay of all these factors contribute to many disease outcomes (12, 113, 114).

Of additional consideration as an important implication of this research, particularly for minority populations, is the potential of stigma related to ADRD diagnosis. Some groups have been found to consider dementia as a normal part of aging (115), while others may find shame in an AD diagnosis or the need to keep such health information private (116–118). We highlight these studies as further evidence of the need to focus research in racially and ethnically diverse communities. Furthermore, we acknowledge that such research should consider both quantitative and qualitative approaches.

This study is not without limitations. First, while we conducted a systematic and structured process for the scoping review, we did not evaluate the quality of the evidence presented or the authors' research methods as part of this review. Second, some studies more clearly identified the characteristics of interest for our review than others, and as such, some of the data presented was left to the interpretation of the authorship team. Third, we acknowledge that there is limited generalizability of our findings to research that has been conducted in the U.S. among racial/ethnic minorities. That said, we find that an important strength of this review is in identifying the knowledge gaps in examining and understanding the genetic factors associated with ADRD among racial/ethnic minority populations, which is of growing disease and economic burden in the U.S.

## Conclusion

Based our findings, we recommend that additional studies be undertaken to map out and more deeply explore ADRD genetic risk factors among racial/ethnic minority populations in the U.S. at levels comparable to non-minority populations. An increased number of larger scale studies of racially/ethnically diverse persons can aid researchers in making more powerful conclusions about genetic associations in ADRD among populations most affected. Examining genetic risk factors for ADRDs in minority populations can deepen our understanding of the interaction between biological or genetic factors and socio-ecological determinants of health. Furthermore, understanding the role of genetic predisposing factors has the potential to increase preventive health measures and screening, which could lead to reduced time to diagnosis and improved ADRD disease management. Lastly, ethical concerns about the impact that this knowledge of genetic risk factors may have on the health and well-being of individuals must be addressed as we continue to obtain more data on these genetic factors. As our population ages and the size of our minority populations increase in the U.S., understanding the burden of ADRD on our aging populations can aid in providing insight into the most appropriate and effective public health actions.

## Data Availability Statement

The original contributions presented in the study are included in the article/supplementary material, further inquiries can be directed to the corresponding author/s.

## Author Contributions

LR conceptualized the study, was a scoping reviewer, and contributed to the manuscript narrative. LI and DF were scoping reviewers, contributed to the manuscript narrative, and helped to edit the manuscript. NR was a scoping reviewer and contributed to the manuscript narrative. BA-C, AR, KL, SU, and QM were scoping reviewers and helped to edit the manuscript. All authors contributed to the article and approved the submitted version.

## Conflict of Interest

The authors declare that the research was conducted in the absence of any commercial or financial relationships that could be construed as a potential conflict of interest.

## Publisher's Note

All claims expressed in this article are solely those of the authors and do not necessarily represent those of their affiliated organizations, or those of the publisher, the editors and the reviewers. Any product that may be evaluated in this article, or claim that may be made by its manufacturer, is not guaranteed or endorsed by the publisher.
